# Anthracycline Cardiotoxicity: A Real‐World Study Based on FDA Adverse Event Reporting System Database

**DOI:** 10.1155/cdr/8073571

**Published:** 2026-08-30

**Authors:** Maoxia Fan, Huimin Lu, Jiacheng Zheng, Yawen Wang, Meng Li, Lijing Zhang

**Affiliations:** ^1^ Dongzhimen Hospital, Beijing University of Chinese Medicine, Beijing, China; ^2^ Shanghai Shuguang Hospital Affiliated to Shanghai Uiversity of traditional Chinese medicine, Shanghai, China; ^3^ China Academy of Chinese Medical Sciences Wangjing Hospital, Beijing, China; ^4^ Beijing University of Chinese Medicine, Beijing, China, bucm.edu.cn

**Keywords:** anthracyclines, cancer, cardiotoxicity, FAERS, pharmacovigilance

## Abstract

**Objective:**

Based on the FDA Adverse Event Reporting System (FAERS) database, data mining of adverse event (AE) signals related to anthracyclines was conducted to explore their potential medication risks, especially cardiotoxicity, in order to promote the rational and safe use of these drugs in clinical practice.

**Methods:**

AE data for anthracyclines (represented by aclarubicin, daunorubicin, doxorubicin, and epirubicin) commonly used to treat hematologic malignancies and solid tumors were retrieved from the FAERS database from January 1, 2004, to June 30, 2025. Disproportionation analysis was primarily used to mine signals of AEs related to anthracyclines.

**Results:**

A total of 324 AE reports related to aclarubicin, 298 related to daunorubicin, 822 related to doxorubicin, and 334 related to epirubicin were extracted from the FAERS database. Based on risk signal detection using the Bayesian confidence propagation neural network (BCPNN) for cardiac AEs: For aclarubicin, left ventricular dysfunction exhibited the strongest risk signal among AEs with positive risk signals, whereas cardiac failure had the largest number of reports. For daunorubicin, supraventricular arrhythmia represented the strongest risk signal among AEs with positive risk signals, and tachycardia had the highest reporting count. For doxorubicin, acute cardiomyopathy showed the strongest risk signal among AEs with positive risk signals, whereas cardiac failure possessed the greatest number of reports. For epirubicin, heart failure with midrange ejection fraction yielded the strongest risk signal among AEs with positive risk signals, and cardiac failure had the largest number of reports.

**Conclusion:**

This study provides a more comprehensive insight into the monitoring, supervision, and management of adverse reactions related to anthracyclines. Clinicians should further focus on the impact of various AEs related to anthracyclines and their corresponding signal strengths, especially the cardiotoxicity of anthracyclines, which is of great value for improving the clinical safety of anthracyclines.

## 1. Introduction

Cancer, as one of the leading causes of death globally, is a significant factor hindering the extension of human life expectancy and causing a substantial disease burden [1]. The discovery of anthracyclines in the 1960s was a major breakthrough in the field of oncology. Despite the revolutionary changes in cancer treatment over the past decades, anthracyclines (including aclarubicin, daunorubicin, doxorubicin, and epirubicin) remain the cornerstone of modern chemotherapy regimens for various cancers [2].

Anthracyclines have been widely used in various solid tumors and hematologic malignancies (including sarcomas, lymphomas, leukemias, and breast cancer) and still hold an important place in standard chemotherapy regimens. Anthracyclines are among the most effective chemotherapeutic agents [2, 3]. The mechanisms by which anthracyclines exert their antitumor effects include (1) inhibition of DNA synthesis, (2) binding to DNA and alkylation, (3) induction of DNA cross‐linking, (4) interference with DNA strand separation and helicase activity, and (5) generation of free radicals and induction of lipid peroxidation [4, 5]. Although highly effective, the clinical use of anthracyclines has long been limited by their significant dose‐dependent cardiotoxicity. Anthracycline cardiotoxicity (ACT) has a high incidence rate and poses great harm, not only limiting the effective implementation of anticancer regimens but also seriously threatening the life safety of cancer patients [6]. The mechanism underlying anthracycline‐induced cardiotoxicity is mainly due to iron‐mediated reactive oxygen species (ROS) generation and oxidative stress caused by anthracyclines. Anthracyclines promote the release of free iron from cardiomyocytes by acting on iron regulatory proteins and transferrin receptors, forming doxorubicin‐Fe^2+^ complexes that accelerate the generation of ROS [7]. Anthracyclines have a cardiophilic nature, which exacerbates oxidative stress in cardiomyocytes [8]. The activation of the oxidative stress system and the gradual accumulation of ROS lead to mitochondrial damage and lipid peroxidation in cardiomyocytes, resulting in irreversible cardiomyocyte injury. ACT manifests primarily as heart failure, arrhythmias, and myocardial injury. The occurrence of ACT leads to a decline in patients′ quality of life and a worsening prognosis, to the extent that the risk of death due to induced cardiotoxicity in cancer patients has now surpassed the risk of cancer recurrence [9]. Therefore, when formulating antitumor chemotherapy regimens, the risk of anthracycline‐induced cardiotoxicity is always a key factor that needs to be carefully considered.

The US FDA Adverse Event Reporting System (FAERS) database is the largest pharmacovigilance repository globally and is a valuable resource for detecting adverse events associated with drug use. Additionally, as the largest spontaneous reporting database of AEs globally, the FAERS database systematically collects and monitors AEs related to approved drugs from pharmaceutical companies, healthcare professionals, and the public [10, 11].

The merits of the current study are manifested in the following aspects. First, by utilizing large‐scale real‐world data extracted from the FAERS database, the present work compensates for the inherent limitations of clinical trials, which are inadequate for identifying rare adverse events and implementing prolonged follow‐up observations. Second, standardized signal detection methodologies, primarily disproportionality analysis, are employed to quantitatively compare the cardiotoxicity risks across various anthracycline‐containing chemotherapy regimens and establish a hierarchical risk order. Third, subgroup analyses are performed to delineate the epidemiological distribution of early‐onset cardiotoxicity stratified by age, sex, and concurrent medications, thereby establishing a foundation for clinical stratified risk management of anthracycline‐related cardiac injury. Furthermore, this study utilizes FAERS data to estimate the incidence risk of cardiotoxicity during anthracycline‐based chemotherapy and compare interregimen discrepancies in the occurrence of early cardiotoxicity. Through pharmacovigilance signal mining based on the FAERS database, we systematically and comprehensively characterize the potential cardiovascular risks of anthracyclines under real‐world clinical settings. The findings of this investigation will serve as a crucial evidence base for the clinical safety evaluation of anthracycline chemotherapeutic agents.

## 2. Methods

### 2.1. Data Sources and Procedures

This retrospective pharmacovigilance study utilized data from the FAERS database (https://fis.fda.gov/extensions/FPD-QDE-FAERS/FPD-QDE-FAERS.html). Reszhouyior fuzzy search, and those with aclarubicin, daunorubicin, doxorubicin, and epirubicin as the primary suspect drugs were selected. The content included the FAERS data files, which contained seven types of datasets—patient demographic and administrative information (DEMO), drug information (DRUG), coded for the adverse events (REAC), patient outcomes (OUTC), report sources (RPSR), therapy start dates and end dates for reported drugs (THER), and indications for drug administration (INDI)—and deleted cases. Although the majority of reports are submitted from the United States, they can be submitted from any country. Therefore, there may be inconsistencies in the names of some drugs and AEs. The symptoms of AEs are coded using the International Medical Dictionary for Regulatory Activities (MedDRA: https://www.meddra.org/), which is an international standardized, clinically validated terminology [12]. MedDRA was used to standardize the system organ class (SOC) and preferred term (PT) of the adverse events collected in this study. The Open Vigil 2.1‐MedDRA (http://h2876314.stratoserver.net:8080/OV21d2/search/) tool was used to search for and extract relevant data in this study [13]. To avoid duplicate reporting, we identified and eliminated duplicate reports from the DEMO table according to the FAERS documentation. In addition, in this study, we included an analysis of restricted indications, selecting only reports submitted by healthcare professionals, including physicians, pharmacists, and other health professionals, to ensure the reliability of the results.

### 2.2. Statistical Analysis

The statistical analysis employed descriptive statistical methods to adverse drug reactions associated with anthracyclines. To find pairs of AEs associated with anthracyclines, we employed several algorithms for metrics. Disproportionation analysis was a tool for hypothesizing possible causal relationships between drugs and adverse reactions, with subsequent clinical assessment of underlying case reports [14]. It included two non‐Bayesian methods—reporting odds ratio (ROR) and the proportional reporting ratio (PRR)—and two Bayesian methods—the Bayesian confidence propagation neural network (BCPNN) and the Empirical Bayesian geometric mean (EBGM) [15]. The advantage of the non‐Bayesian method is simple to calculate and has high sensitivity, but when the number of adverse events is small, the likelihood of false positives is high [16]. The Bayesian approach is stable. It accounts for the uncertainty in the disproportionate rate when the reports are small, reduces the likelihood of false positives, and is used for pattern recognition in higher dimensions, but it is computationally complex and has a relatively lagged signal detection time [17]. The meanings of a, b, c, and d were shown in Table 1. Therefore, in this study, we used all the four methods to investigate the potential signals between anthracyclines and AEs. Signals that satisfy all four algorithms at the same time were considered statistically significant. The calculation formulas and signal detection criteria are detailed in Table 2 [18]. The higher the signal value, the stronger the risk signal, indicating a higher increased risk of PT occurrence [13]. In addition, a gender disambiguation analysis was also performed to further explore gender differences in drug‐related AEs.

**Table 1:** Four‐fold table of disproportionality methods.

Item	Number of target adverse event reports	Number of other adverse event reports	Total
Target drug	*a*	*b*	*a* + *b*
Other drugs	*c*	*d*	*c* + *d*
Total	*a* + *c*	*b* + *d*	*N* = *a* + *b* + *c* + *d*

**Table 2:** Summary of four signal detection algorithms.

Methods	Formula	Threshold value
ROR	*R* *O* *R* = (*a* *d*)/(*b* *c*)	*a* ≥ 3; A signal is generated if the lower limit of 95%Cl of ROR > 1.
	$95\%\text{CI}=e^{\text{In}\left(ROR\right)\pm 1.96}\sqrt{\frac{1}{a}+\frac{1}{b}+\frac{1}{c}+\frac{1}{d}}$	
PRR	*P* *R* *R* = [*a*(*c* + *d*)]/[*c*(*a* + *b*)]	*a* ≥ 3; PRR ≥ 2, *χ* ^2^ ≥ 4, a signal is generated.
	$\chi^{2}=\frac{\left(a+b+c+d\right){\left(ad-bc\right)}^{2}}{\left(a+b\right)\left(c+d\right)\left(a+c\right)\left(b+d\right)}$	
BPCNN	$IC=\log_{2}\frac{a\left(a+b+c+d\right)}{\left(a+b\right)\left(a+c\right)}$	If IC025 > 0 (IC025: the lower bound of 95% CI), the BPCNN is a positive signal. The signal intensity was strikingly correlated with the IC025 value.
	$952\%\text{CI}=E\left(IC\right)\pm \times \sqrt{V\left(IC\right)}$	
EBGM	*E* *B* *G* *M* = (*aN*)/[(*a* + *b*)(*a* + *c*)]	If EBGM05 > 2 (EBGM05: the lower bound of 95% CI), the BPCNN is a striking signal.
	$95\%\text{CI}=e^{\text{In}\left(EGBM\right)\pm 1.96}\sqrt{\frac{1}{a}}+\frac{1}{b}+\frac{1}{c}+\frac{1}{d}$	

Abbreviations: BCPNN, Bayesian confidence propagation neural network; EBGM, Empirical Bayes geometric mean; PRR, proportional reporting ratio; ROR, reporting odds ratio.

All statistical analyses and visualizations were conducted using R software (https://www.r-project.org/; version 4.0.0).

## 3. Results

### 3.1. Descriptive Analysis‐Characteristics of AE Reports Related to Anthracyclines

**Figure 1:**
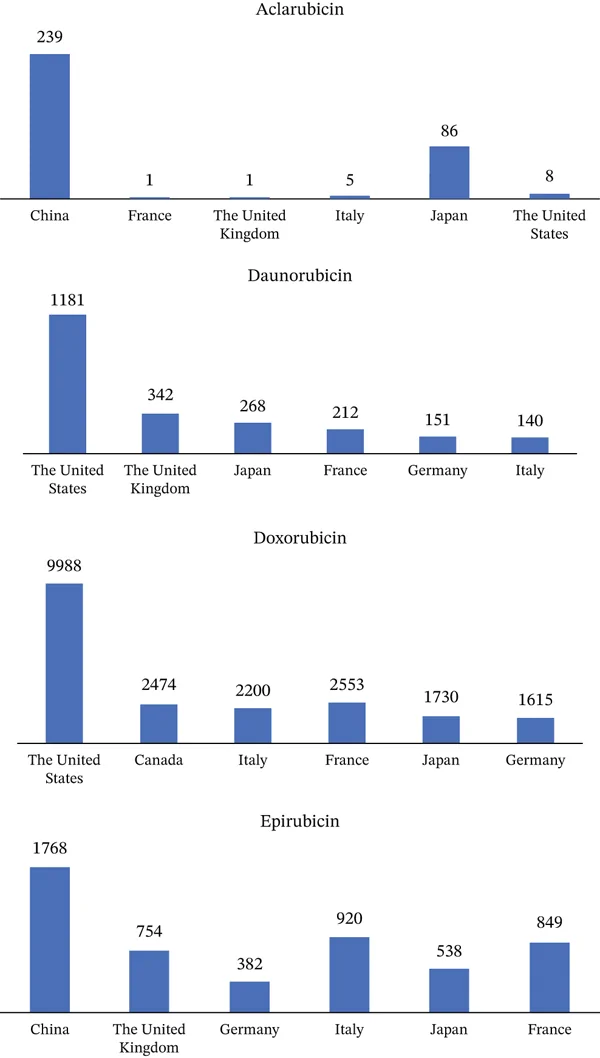
TOP 6 reporter country.

**Figure 2:**
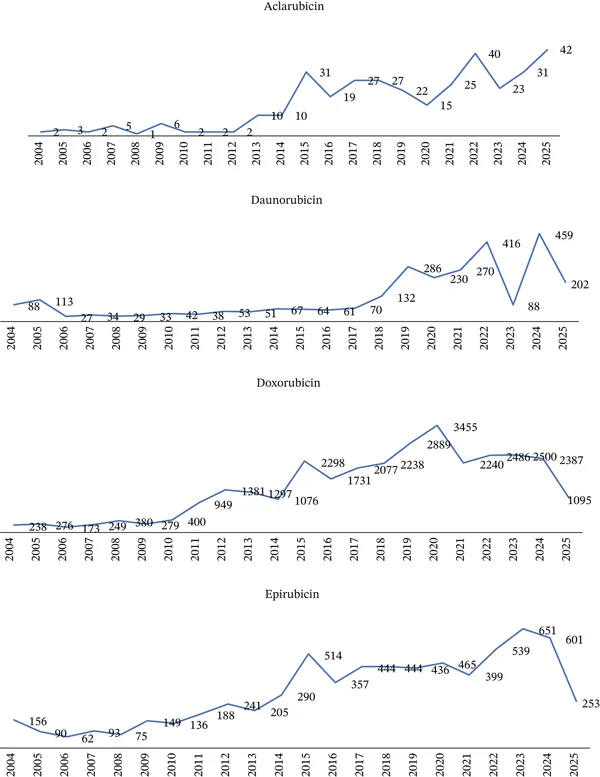
Distribution of anthracyclines reporting year.

AE reports related to aclarubicin, daunorubicin, doxorubicin, and epirubicin were obtained from the FAERS database (from the first quarter of 2004 to the second quarter of 2025). After excluding irrelevant and duplicate reports, a total of 324 AE reports related to aclarubicin, 298 AE reports related to daunorubicin, 822 AE reports related to doxorubicin, and 334 AE reports related to epirubicin were extracted from the FAERS database. Detailed reports on AEs of anthracyclines are provided in supporting information 1. The clinical characteristics of patients with AEs of anthracyclines are shown in Table 3:

1. In the AE reports related to aclarubicin, patients aged 18–65 years accounted for over half of the cases, at 51.9%, and males were more represented than females. The reporters were mainly physicians, pharmacists, and other health professionals, accounting for 64.6%. In this study, the most severe outcome among multiple choices was selected as the final outcome. The most common outcome was death, with a total of 63 cases, accounting for 18.2%, followed by hospitalization or prolonged hospital stay, with a total of 49 cases, accounting for 14.1%. As shown in Figure 1, the top six countries in terms of the number of reports were China, Japan, the United States, Italy, the United Kingdom, and France. As shown in Figure 2, the number of adverse events reported each year was relatively low, reaching a peak in 2025 with 40 cases reported. However, only the second quarter of 2025 was included in the statistics, and subsequent AE reports still need to be counted.
2. In the AE reports related to daunorubicin, patients aged 18–65 years accounted for a significant proportion (31.9%), with males being the majority, representing 44.1%. The reports were mainly submitted by physicians, pharmacists, and other health experts, accounting for 60.6%. In this study, the most severe outcome among multiple choices was selected as the final outcome. The most common outcome was death, with a total of 599 cases, accounting for 21.0%, followed by hospitalization or prolonged hospital stay, with a total of 590 cases, accounting for 20.7%. As shown in Figure 1, the top six countries in terms of the number of reports were the United States, the United Kingdom, Japan, France, Germany, and Italy. As shown in Figure 2, the number of adverse events reported each year showed a slow fluctuating increase, with a sharp rise to 132 cases in 2018, followed by large fluctuations in subsequent years. The highest number of reports was in 2024, with 459 cases. However, only the second quarter of 2025 was included in the statistics, and subsequent AE reports still need to be counted.
3. In the AE reports related to doxorubicin, patients aged 18–65 years accounted for a significant proportion, at 39.2%, with females being the majority, representing 46.0%. The reports were mainly submitted by physicians, pharmacists, and other health experts, accounting for 69.4%. In this study, the most severe outcome among multiple choices was selected as the final outcome. The most common outcome was hospitalization or prolonged hospital stay, with a total of 6536 cases, accounting for 20.4%, followed by death, with a total of 5078 cases, accounting for 15.8%. As shown in Figure 1, the top six countries in terms of the number of reports were the United States, France, Canada, Italy, Japan, and Germany. As shown in Figure 2, the number of adverse events reported each year showed a fluctuating increase, reaching a peak in 2020 with 3455 cases, followed by a downward trend. However, only the second quarter of 2025 was included in the statistics, and subsequent AE reports still need to be counted.
4. In the AE reports related to epirubicin, patients aged 18–65 years accounted for over half of the cases, at 57.0%, with females being the majority, representing 70.2%. The reports were mainly submitted by physicians, pharmacists, and other health experts, accounting for 60.0%. In this study, the most severe outcome among multiple choices was selected as the final outcome. The most common outcome was hospitalization or prolonged hospital stay, with a total of 1744 cases, accounting for 25.7%, followed by death, with a total of 563 cases, accounting for 8.3%. As shown in Figure 1, the top six countries in terms of the number of reports were China, Italy, France, the United Kingdom, Japan, and Germany. As shown in Figure 2, the number of adverse events reported each year showed a fluctuating increase, reaching a peak in 2023 with 651 cases. There was a slight decrease in 2024. However, only the second quarter of 2025 was included in the statistics, and subsequent AE reports still need to be counted.

**Table 3:** The clinical characteristics of patients with adverse events of anthracyclines.

Aclarubicin		Daunorubicin		Doxorubicin		Epirubicin	
**X.**	**Overall (** **N** = 347**)**	**X.**	**Overall (** **N** = 2853**)**	**X.**	**Overall (** **N** = 32094**)**	**X.**	**Overall (** **N** = 6788**)**
**Sex**	**N**	**Sex**	**N**	**Sex**	**N**	**Sex**	**N**
F	122 (35.2%)	F	1017 (35.6%)	F	14748 (46.0%)	F	4762 (70.2%)
M	147 (42.4%)	M	1258 (44.1%)	M	10295 (32.1%)	M	941 (13.9%)
Missing	78 (22.5%)	Missing	578 (20.3%)	Missing	7051 (22.0%)	Missing	1085 (16.0%)
**Age**	**N**	**Age**	**N**	**Age**	**N**	**Age**	**N**
< 18 years	20 (5.8%)	< 18 years	350 (12.3%)	< 18 years	2907 (9.1%)	< 18 years	146 (2.2%)
> 85 years	1 (0.3%)	> 85 years	3 (0.1%)	> 85 years	131 (0.4%)	> 85 years	7 (0.1%)
18~64.9 years	180 (51.9%)	18~64.9 years	909 (31.9%)	18~64.9 years	12588 (39.2%)	18~64.9 years	3868 (57.0%)
65~85 years	61 (17.6%)	65~85 years	610 (21.4%)	65~85 years	6208 (19.3%)	65~85 years	1168 (17.2%)
Missing	85 (24.5%)	Missing	981 (34.4%)	Missing	10260 (32.0%)	Missing	1599 (23.6%)
**Outcome**	**N**	**Outcome**	**N**	**Outcome**	**N**	**Outcome**	**N**
Death	63 (18.2%)	Death	599 (21.0%)	Congenital anomaly	70 (0.2%)	Congenital anomaly	12 (0.2%)
Disability	5 (1.4%)	Disability	5 (0.2%)	Death	5078 (15.8%)	Death	563 (8.3%)
Hospitalization	49 (14.1%)	Hospitalization	590 (20.7%)	Disability	117 (0.4%)	Disability	110 (1.6%)
Life‐threatening	28 (8.1%)	Life‐threatening	170 (6.0%)	Hospitalization	6536 (20.4%)	Hospitalization	1744 (25.7%)
Other	202 (58.2%)	Other	1489 (52.2%)	Life‐threatening	1769 (5.5%)	Life‐threatening	304 (4.5%)
				Other	18524 (57.7%)	Other	4055 (59.7%)
**Reporter type**	**N**	**Reporter type**	**N**	**Reporter type**	**N**	**Reporter type**	**N**
Consumer	23 (6.6%)	Consumer	442 (15.5%)	Consumer	1609 (5.0%)	Consumer	677 (10.0%)
Health professional	105 (30.3%)	Health professional	483 (16.9%)	Health professional	7142 (22.3%)	Health professional	1272 (18.7%)
Pharmacist	2 (0.6%)	Pharmacist	139 (4.9%)	Pharmacist	1850 (5.8%)	Pharmacist	514 (7.6%)
Physician	117 (33.7%)	Physician	1108 (38.8%)	Physician	13264 (41.3%)	Physician	2287 (33.7%)
Missing	100 (28.8%)	Missing	681 (23.9%)	Missing	8229 (25.6%)	Missing	2038 (30.0%)

### 3.2. SOCs Involved in AEs Related to Anthracyclines

For the 49 positive risk signals of AEs related to aclarubicin, they were classified and merged according to SOCs, basically covering the SOCs of adverse reactions recorded in the aclarubicin instructions. However, no AEs related to the urinary system and psychiatric disorders mentioned in the instructions were screened out. In addition, six SOCs not mentioned in the aclarubicin instructions were mined—endocrine disorders; infections and infestations; investigations; neoplasms benign, malignant, and unspecified (including cysts and polyps); respiratory, thoracic, and mediastinal disorders; and injury, poisoning, and procedural complications.

For the 289 positive risk signals of AEs related to daunorubicin, they were classified and merged according to SOCs, basically covering the SOCs of adverse reactions recorded in the daunorubicin instructions. However, no AEs related to reproductive system and breast disorders mentioned in the instructions were screened out. In addition, six SOCs not mentioned in the daunorubicin instructions were mined—congenital, familial, and genetic disorders; endocrine disorders; musculoskeletal and connective tissue disorders; nervous system disorders; psychiatric disorders; and injury, poisoning, and procedural complications.

For the 814 positive risk signals of AEs related to doxorubicin, they were classified and merged according to SOCs, covering the SOCs of adverse reactions recorded in the doxorubicin instructions. In addition, 11 SOCs not mentioned in the doxorubicin instructions were mined—congenital, familial, and genetic disorders; endocrine disorders; eye disorders; immune system disorders; investigations; musculoskeletal and connective tissue disorders; neoplasms benign, malignant and unspecified (including cysts and polyps); pregnancy, puerperium, and perinatal conditions; psychiatric disorders; reproductive system and breast disorders; and injury, poisoning, and procedural complications.

For the 331 positive risk signals of AEs related to epirubicin, they were classified and merged according to SOCs, basically covering the SOCs of adverse reactions recorded in the epirubicin instructions. However, no AEs related to the immune system mentioned in the instructions were screened out. In addition, seven SOCs not mentioned in the epirubicin instructions were mined—congenital, familial, and genetic disorders; endocrine disorders; hepatobiliary disorders; musculoskeletal and connective tissue disorders; nervous system disorders; pregnancy, puerperium, and perinatal conditions; and psychiatric disorders.

**Figure 3:**
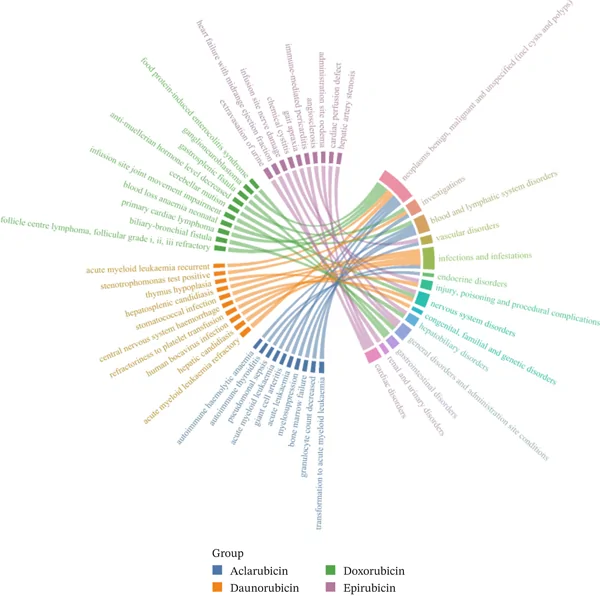
SOC distribution of the Top 10 PTs with the highest signal intensity for the anthracyclines.

Figure 3 shows the SOC distribution of the Top 10 PTs with the highest signal intensity for the anthracyclines. Detailed data are provided in supporting information 1.

### 3.3. Cardiotoxicity Related to Anthracyclines

**Figure 4:**
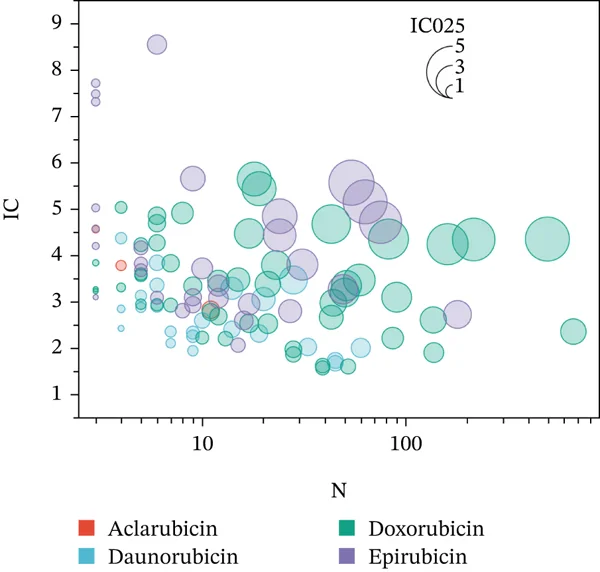
Bubble plot of the signal intensity and reporting frequency of PTs of cardiotoxicity associated with anthracyclines.

As shown in Table 4, data are as follows: (1) Among the 49 positive risk signal AEs related to aclarubicin, three cardiovascular‐related AEs were mined. Ranked in descending order of risk signal strength by the BCPNN, left ventricular (LV) dysfunction had the strongest risk signal. Ranked in descending order of the number of reports, cardiac failure was at the top. (2) Among the 289 positive risk signal AEs related to daunorubicin, 23 cardiovascular‐related AEs were mined. Ranked in descending order of risk signal strength by the BCPNN, arrhythmia supraventricular had the strongest risk signal. Ranked in descending order of the number of reports, tachycardia was at the top. (3) Among the 814 positive risk signal AEs related to doxorubicin, 50 cardiovascular‐related AEs were mined. Ranked in descending order of risk signal strength by the BCPNN, cardiomyopathy acute had the strongest risk signal. Ranked in descending order of the number of reports, cardiac failure was at the top. (4) Among the 331 positive risk signal AEs related to epirubicin, 31 cardiovascular‐related AEs were mined. Ranked in descending order of risk signal strength by the BCPNN, heart failure with midrange ejection fraction (EF) had the strongest risk signal. Ranked in descending order of the number of reports, cardiac failure was at the top. Figure 4 presents a bubble plot showing the signal intensity and reporting frequency of PTs related to cardiotoxicity of anthracyclines.

**Table 4:** The PTs with the strongest risk signals and highest reported incidence of cardiotoxicity associated with anthracyclines.

Anthracyclines	PT	N	IC (IC025)
Aclarubicin	Cardiac failure	11	2.85 (1.41)
	Left ventricular dysfunction	3	4.58 (0.38)
Daunorubicin	Tachycardia	60	2.03 (1.59)
	Arrhythmia supraventricular	4	4.39 (0.78)
Doxorubicin	Cardiac failure	663	2.38 (2.26)
	Cardiomyopathy acute	18	5.66 (3.12)
Epirubicin	Cardiac failure	179	2.75 (2.48)
	Heart failure with midrange ejection fraction	6	8.55 (1.61)

The cardiotoxicity of anthracyclines is specifically demonstrated in supporting information 2.

### 3.4. Sensitivity Analysis—Cardiotoxicity Related to Anthracyclines by Gender

**Figure 5:**
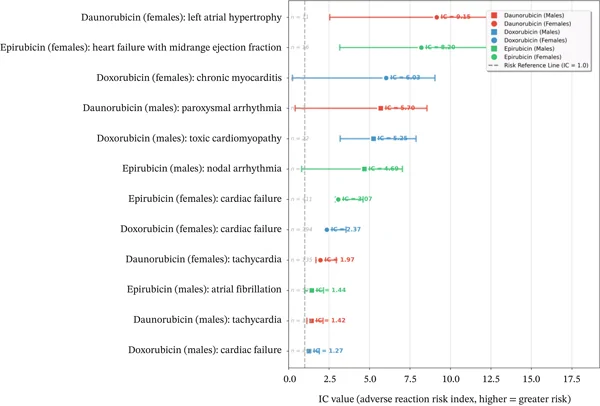
The PTs with the strongest risk signals and highest reported incidence of cardiotoxicity associated with anthracyclines by gender.

To further verify the robustness of our research results, we conducted a sensitivity analysis. Given the different proportions of males and females, we performed risk signal detection for cardiovascular AEs related to anthracyclines separately by gender. The results were ranked according to the risk signal strength detected by the BCPNN. As shown in Figure 5, data are as follows:

1. For aclarubicin, no positive cardiovascular adverse reaction risk signals were found in either male or female populations according to the results of the four risk signal detection methods.
2. For daunorubicin in the male population, ranked in descending order of risk signal strength by the BCPNN, paroxysmal arrhythmia had the strongest risk signal. Ranked in descending order of the number of reports, tachycardia was at the top. In the female population, ranked in descending order of risk signal strength by the BCPNN, left atrial hypertrophy had the strongest risk signal. Ranked in descending order of the number of reports, tachycardia was at the top.
3. For doxorubicin in the male population, ranked in descending order of risk signal strength by the BCPNN, toxic cardiomyopathy had the strongest risk signal. Ranked in descending order of the number of reports, cardiac failure was at the top. In the female population, ranked in descending order of risk signal strength by the BCPNN, chronic myocarditis had the strongest risk signal. Ranked in descending order of the number of reports, cardiac failure was at the top.
4. For epirubicin in the male population, ranked in descending order of risk signal strength by the BCPNN, nodal arrhythmia had the strongest risk signal. Ranked in descending order of the number of reports, atrial fibrillation (AF) was at the top. In the female population ranked in descending order of risk signal strength by the BCPNN, heart failure with midrange EF had the strongest risk signal. Ranked in descending order of the number of reports, cardiac failure was at the top.

Detailed results are shown in the supporting information 3.

## 4. Discussion

Anthracyclines are the cornerstone of contemporary chemotherapy regimens for various cancers. Although highly effective, the use of anthracyclines is limited by significant dose‐dependent cardiotoxicity. This cardiotoxicity caused by anthracyclines severely affects the quality of life and overall survival rate of patients undergoing chemotherapy for cancer, and it is dose‐dependent, progressive, and irreversible [19]. In this study, based on data from the FAERS database, we identified and summarized the occurrence of multiple adverse events following chemotherapy with anthracycline‐containing regimens—essentially covering many of the adverse reactions mentioned in the instructions, and analyzed those that are prone to be associated with cardiac diseases—to enhance the understanding of the safety of anthracyclines among clinical staff.

Analysis of results showed that the population experiencing adverse events related to anthracyclines is mainly concentrated in the age group of 18–85 years. The groups experiencing adverse reactions related to aclarubicin and daunorubicin are predominantly male, whereas those experiencing adverse reactions related to doxorubicin and epirubicin are predominantly female. The highest number of reports related to aclarubicin and epirubicin comes from China, whereas the highest number of reports related to daunorubicin and doxorubicin comes from the United States. The most common severe adverse reaction outcome related to aclarubicin and daunorubicin is death, while the most common severe adverse reaction outcome related to doxorubicin and epirubicin is hospitalization or prolonged hospital stay. The long‐term cardiovascular outcomes of cancer survivors vary due to multiple risk factors. In the AE reports, the majority of reporters are physicians and other health experts, which may be related to the trust patients have in doctors, enabling them to promptly report drug‐related adverse reactions to physicians. This encourages clinical pharmacists to actively participate in the entire process of patient medication, enhance medication monitoring for patients, pay attention to adverse drug reactions, and identify problems in a timely manner to provide corresponding recommendations, thereby further improving the safety of clinical drug use. It also indirectly reflects the authenticity and accuracy of the reports.

According to the BCPNN risk signal detection, the strongest risk signal for aclarubicin‐related adverse reactions is transformation to acute myeloid leukemia; for daunorubicin‐related adverse reactions, the strongest risk signal is blood copper increased; for doxorubicin‐related adverse reactions, the strongest risk signal is follicle center lymphoma, follicular Grades i, ii, and iii refractory; and for epirubicin‐related adverse reactions, the strongest risk signal is extravasation of urine. In addition, the AEs related to anthracyclines involve SOCs, and SOCs not mentioned in the instructions were mined. The newly mined adverse reactions and SOCs that have not been included in the instructions are worth paying attention to, especially the PTs with strong positive risk signals.

### 4.1. Cardiotoxicity Related to Anthracyclines

Among the positive risk signal AEs related to aclarubicin, LV dysfunction had the strongest risk signal, whereas cardiac failure had the highest number of reports. Among the positive risk signal AEs related to daunorubicin, arrhythmia had the strongest risk signal, whereas tachycardia had the highest number of reports. Among the positive risk signal AEs related to doxorubicin, cardiomyopathy had the strongest risk signal, whereas cardiac failure had the highest number of reports. Among the positive risk signal AEs related to epirubicin, heart failure with midrange EF had the strongest risk signal, whereas cardiac failure had the highest number of reports. In the sensitivity analysis, it is worth noting that we found differences in the risk signal strength and the number of reports of anthracycline‐related cardiac AEs between male and female populations.

Cardiotoxicity is the most serious adverse reaction of anthracyclines. The main mechanism of anthracycline‐induced cardiotoxicity is the generation of iron‐mediated ROS and the promotion of oxidative stress in cardiomyocytes. After chelating iron ions, anthracyclines trigger the production of oxygen radicals, especially hydroxyl radicals, leading to lipid peroxidation of cardiomyocyte membranes and damage to mitochondrial DNA in the heart [20, 21]. Compared with other cells, anthracyclines have a cardiophilic nature and are more likely to stay in cardiomyocytes. Cardiac tissue lacks catalase and has weaker antioxidant activity. In addition, cardiomyocytes are rich in mitochondria, which are the source of ROS production. Anthracyclines have a high affinity for cardiolipin, can enter mitochondria, and bind to cardiolipin to inhibit the respiratory chain, causing cardiac damage [22]. Clinical studies and observations have shown that cardiotoxicity caused by anthracyclines is mostly progressive and irreversible, especially when anthracyclines are used for the first time, which can easily cause cardiac damage. Therefore, it is particularly important to actively focus on cardiotoxicity caused by anthracyclines, which provides a basis for clinical prevention.

Existing research has found that cardiotoxicity initially manifests in adult cancer patients as clinical congestive heart failure (CHF), characterized by pulmonary edema, fluid overload, and decreased exercise tolerance. This phenomenon was first reported by Von Hoff et al. in 1979 [23], with an overall incidence rate of 2.2%. The cumulative doxorubicin dose‐dependent CHF incidence rates were 3%, 7%, and 18% in the 400, 550, and 700 mg/m^2^ dose groups, respectively. A retrospective pooled analysis of three breast cancer and lung cancer trials by Swain et al. [24] showed higher CHF incidence rates—4.7%, 26%, and 48% in the 400, 550, and 700 mg/m^2^ dose groups, respectively—which provides the current basis for limiting lifetime cumulative doxorubicin equivalent exposure to ≤ 450 mg/m^2^ in patients without a history of chest radiotherapy. Further studies on biopsy‐confirmed doxorubicin‐induced cardiomyopathy showed a poor prognosis—with a hazard ratio of 3.46 for fatal cardiotoxicity and a two‐year mortality rate of 50%—similar to restrictive and infiltrative cardiomyopathies [25]. Subclinical cardiotoxicity is usually defined as asymptomatic left ventricular systolic dysfunction (LVSD) in cardiac imaging, with a decrease in LV EF of > 10 percentage points to EF<50% [26, 27]. When detected as an early biomarker of cancer cardiotoxicity, 21%–40% of patients had elevated troponins after anthracycline chemotherapy, regardless of the detection method used [28–30]. Although troponin‐I and troponin‐T levels cannot be directly compared using current detection methods, representative levels were 11–120 ng/L in low‐dose anthracycline regimens and from 160 to over 1900 ng/L in high‐dose regimens [31]. Cancer itself may be arrhythmogenic, and its treatment may increase the incidence of supraventricular tachycardia and AF [32, 33]. A large cohort study of 24,125 cancer patients showed that the baseline incidence of AF was 2.4%, and the incidence of new‐onset AF was 1.8% [34]. Early short‐term arrhythmia studies recorded that 3% of patients had ventricular ectopy 1 h after infusion, which increased to 24% after 1–24 h of Holter monitoring.

### 4.2. In‐Depth Analysis and Discussion on Risk Signals of Cardiotoxicity Related to Anthracyclines

#### 4.2.1. Aclarubicin: LV Dysfunction as the Intrinsic Association With the Strongest Risk Signal

##### 4.2.1.1. Specificity of Drug Action Target

Aclarubicin exerts moderate selective inhibition on cardiac topoisomerase II*β*, primarily damaging structures related to myocardial contraction and mitochondrial function. It preferentially induces LV systolic/diastolic dysfunction rather than electrophysiological injuries such as arrhythmias, which constitutes the structural pharmacological basis for LV dysfunction being its strongest signal.

##### 4.2.1.2. Intrinsic Uniformity of Clinical Phenotypes

LV dysfunction and heart failure belong to the same spectrum of myocardial structural and contractile impairment. Distinct from the electrophysiological abnormalities caused by daunorubicin, this confirms that the cardiotoxicity of aclarubicin is characterized by organic cardiac dysfunction as its core feature. Differences in signal intensity and report count merely reflect distinct disease stages, whereas the pathological essence is highly uniform [16].

#### 4.2.2. Daunorubicin: The Mechanism Underlying Supraventricular Arrhythmia as the Strongest Risk Signal

##### 4.2.2.1. Selective Inhibition of Ion Channels

Daunorubicin can specifically block myocardial potassium channels (Ikr, Ito) and sodium channels, prolonging the action potential duration and triggering abnormal automaticity in atrial myocytes. This significantly increases the risk of supraventricular arrhythmias such as atrial premature beats, atrial flutter, and AF, forming the electrophysiological basis for its strongest risk signal.

##### 4.2.2.2. Acute Autonomic Nervous System Dysfunction

Daunorubicin can rapidly activate the sympathetic nerve and inhibit the vagus nerve, resulting in an increased atrial rate and abnormal atrioventricular conduction, directly inducing supraventricular arrhythmias and tachycardia. This effect has a rapid onset and high incidence, making it the most prominent positive signal in pharmacovigilance [16].

#### 4.2.3. Doxorubicin: Biological Mechanisms Uniquely Associated With Acute Cardiomyopathy

##### 4.2.3.1. Iron‐Mediated Oxidative Stress Burst Injury

Doxorubicin forms a complex with iron ions, generating a large amount of ROS within a short period. This directly leads to lipid peroxidation of the myocardial cell membrane and mitochondrial DNA fragmentation, causing acute edema and necrosis of cardiomyocytes, and rapidly inducing acute cardiomyopathy.

##### 4.2.3.2. Irreversible Inhibition of TopoII*β*

Doxorubicin exhibits high affinity and potent inhibition of cardiac TopoII*β*, completely blocking mitochondrial DNA replication and transcription, resulting in a sharp decline in ATP synthesis and acute failure of myocardial energy metabolism, which constitutes the core molecular mechanism of acute cardiomyopathy.

##### 4.2.3.3. Acute Collapse of Calcium Homeostasis

Inhibition of SERCA2a activity leads to intracellular calcium overload and acute loss of myocardial contractile function, progressing to acute cardiomyopathy within days to weeks—much faster than the chronic injury induced by other anthracyclines.

##### 4.2.3.4. Pharmacokinetic Characteristics

Doxorubicin has a high myocardial distribution concentration and a long elimination half‐life, reaching the toxic threshold rapidly. This is the key reason why its risk of acute cardiomyopathy is significantly higher than that of other anthracyclines [35].

#### 4.2.4. Epirubicin: Expansion of Moderate Reduction in EF as a Sign of Heart Failure (HFmrEF)

##### 4.2.4.1. High Consistency With Existing Knowledge

As an epimer of doxorubicin, epirubicin shows low myocardial accumulation and mild toxicity. It is more likely to induce moderate cardiac dysfunction rather than severe heart failure. The finding that HFmrEF represents the strongest signal is consistent with its toxicity profile.

##### 4.2.4.2. Important Expansion of Existing Knowledge

The first identification of the specific heart failure phenotype of epirubicin breaks the homogeneous cognition of anthracycline‐induced heart failure phenotypes and confirms that HFmrEF is the most specific cardiotoxicity marker of epirubicin, providing a target for precise clinical monitoring.

##### 4.2.4.3. Establishment of HFmrEF as an Early Warning Node

It suggests that close monitoring of the LVEF range of 41%–49% is required after epirubicin administration, allowing early intervention to block progression to HFrEF and optimize cardioprotective strategies.

### 4.3. Gender Differences in Cardiotoxicity Associated With Anthracyclines

#### 4.3.1. Core Role of the Iron Metabolism Pathway

Females have lower hepcidin levels and higher myocardial free iron content. Because anthracycline‐induced toxicity is highly dependent on iron‐mediated ROS production, the iron metabolic profile in females significantly amplifies oxidative damage, resulting in risk signal intensity and report counts distinct from those in males.

#### 4.3.2. Gender Differences in the Oxidative Stress Pathway

Estrogen regulates the expression of antioxidant enzymes (SOD and GSH‐Px), conferring certain antioxidant protection in premenopausal females, which is lost after menopause. In contrast, male androgens promote ROS generation, further exacerbating the gender disparity in susceptibility [16].

#### 4.3.3. Gender Specificity of Drug‐Metabolizing Enzymes

Females exhibit lower activity of metabolic enzymes such as CYP450, leading to longer myocardial retention of toxic anthracycline metabolites. This acts synergistically with the iron metabolism–oxidative stress axis to amplify gender differences in cardiotoxicity [35, 36].

#### 4.3.4. Mutual Validation of Clinical and Mechanistic Evidence

The gender‐specific findings of this study are consistent with clinical observations of a higher incidence of anthracycline‐induced cardiotoxicity in females. Mechanistically, we verified that the iron metabolism–oxidative stress axis constitutes the core mechanism underlying gender differences, providing a theoretical basis for gender‐specific prevention and treatment strategies [35].

### 4.4. Limitations

The FAERS database provides potential drug risks for clinical safe medication use and can improve the safety of drug use. However, the FAERS database itself has the following limitations. First, the voluntary reporting in FAERS may be affected by various factors, such as the awareness of the adverse reaction and the emphasis on its occurrence, which may lead to underreporting or overreporting of AEs [37]. Second, disproportionality analysis only provides a statistical assessment of signal strength, not actual risk. Third, the causal relationship between drugs and AEs does not need to be confirmed in the FAERS database. Some AE reports may have incomplete clinical and medical information, such as age in this study. They usually do not include detailed information for evaluating AEs. It is not easy to distinguish whether the risk signal is due to the underlying disease or the adverse reaction of the drug, such as heart failure and AF. Therefore, the involvement of drug AEs in the system organ may be affected by the patient′s underlying diseases. Thus, this causal relationship cannot be established and needs further validation [38].

However, this study also has the following limitations: Absence of dose‐response analysis prevents assessment of whether signals correlate with cumulative anthracycline exposure; Lack of adjustment for concomitant cardiotoxic medications (e.g., trastuzumab and radiation) confounds signal interpretation; No consideration of time‐to‐event data limits understanding of whether signals represent acute or chronic toxicity; The requirement that signals satisfy all four algorithms simultaneously may be overly conservative, potentially missing clinically important signals with lower reporting frequencies.

In addition, although this study identified gender‐based disparities in cardiotoxicity signals, the inherent limitations of the FAERS database precluded a thorough investigation of potential confounding factors. Key constraints include the inability to adjust for intergender differences in cumulative drug exposure; failure to account for critical covariates such as age distribution, tumor histology, and concomitant medications; and the inability to determine whether the observed gender differences reflect genuine biological variation or reporting bias. Prospective clinical and basic studies are warranted to validate the adverse events identified in this study.

## 5. Conclusion

In this study, pharmacovigilance analysis of anthracycline‐related adverse reactions, especially cardiotoxicity, was conducted using the FAERS database. Disproportionality analysis is of great value in pharmacovigilance research. This study used four algorithms to detect risk signals at the PT level, verified and supplemented AEs not mentioned in the instructions, and found differences in adverse reactions, cardiotoxicity, and SOCs related to the four representative anthracyclines, which can provide references for clinical individualized treatment. This study provides evidence for the further rational and safe use of anthracyclines in clinical practice.

## Author Contributions

M.F., Y.W., and J.Z. participated in the research design. M.F., Y.W., and L.Z. conducted a literature search and screened data extraction. M.F., J.Z., and L.Z. analyzed the data, did the statistical analysis, and wrote the manuscript. M.F., H.L., and M.L. participated in the correction of the manuscript. M.F. and H.L. are the cofirst authors.

## Funding

This work was supported by the National Key Science and Technology Project of China: Study on Intervention Scheme and Mechanism of Traditional Chinese Medicine for Prevention and Treatment of Anthracycline‐ËœRelated Cardiac Injury (Grant No. SQ2023AAA032386), A Clinical Study of Chinese Materia Medica for Preventing anthracycline‐induced cardiotoxicity and Replenishing Qi‐Nourishing Yin Therapy for Anthracycline‐Related Frequent Ventricular Premature Beats (Grant No. 2023ZD0502604), and the National Key Research and Development Program: Research on Innovative Pathogenesis and Clinical Diagnosis and Treatment Strategies for Coronary Heart Disease‐Related Heart Failure (No. 2022YFC3500101).

## Disclosure

All authors reviewed the manuscript. All authors read and approved the final version of the manuscript.

## Ethics Statement

The data for this study were derived from public databases and therefore no ethical review and approval are required.

## Conflicts of Interest

The authors declare no conflicts of interest.

## Supporting Information

Additional supporting information can be found online in the Supporting Information section.

## Supporting information


**Supporting Information 1** File S1: 

**Supporting Information 2** File S2: 

**Supporting Information 3** File S3: Table S1: The signal strength and reporting quantity of cardiotoxicity related to daunorubicin in males. Table S2: The signal strength and reporting quantity of cardiotoxicity related to daunorubicin in females. Table S3: The signal strength and reporting quantity of cardiotoxicity related to doxorubicin in males. Table S4: The signal strength and reporting quantity of cardiotoxicity related to doxorubicin in females. Table S5: The signal strength and reporting quantity of cardiotoxicity related to epirubicin in males. Table S6: The signal strength and reporting quantity of cardiotoxicity related to epirubicin in females.

## Data Availability

All relevant data are within the manuscript and its supporting information.
